# Comparison of the EQ-5D-3L and EQ-5D-5L instruments in patients undergoing unicompartmental knee arthroplasty

**DOI:** 10.3389/fmed.2024.1451979

**Published:** 2025-01-06

**Authors:** Zhipeng Tai, Dongping Wan, Qiang Zan, Yuanchi Huang, Chao Xu

**Affiliations:** ^1^Department of Knee Joint Surgery, Honghui Hospital, Xi’an Jiaotong University, Xi’an, China; ^2^The First Clinical Medical College, Shaanxi University of Chinese Medicine, Xianyang, China; ^3^Guangxi University of Chinese Medicine, Nanning, China

**Keywords:** EQ-5D-3L, EQ-5D-5L, comparing, patient-reported outcome measures, unicompartmental knee arthroplasty

## Abstract

**Purpose:**

The purpose of this investigation is to assess and contrast the effectiveness of the two EuroQol five dimensions questionnaire (EQ-5D) versions—EQ-5D-3L and EQ-5D-5L—in assessing one-year quality of life outcomes for patients with knee osteoarthritis (KOA) undergoing unicompartmental knee arthroplasty (UKA).

**Material and method:**

From the medical records at the Honghui Hospital, Xi’an Jiaotong University, 402 individuals aged 50 and above, who were one-year post-operation, were selected to fill out survey questionnaires during their return hospital visits. Of these, 231 respondents (57.5%) completed the questionnaire; 228 completed both versions, and 56 completed the EQ-5D retest questionnaire. The assessment included missing data, ceiling effects, informativity and discriminatory power, as well as response consistency, redistribution properties, and inconsistency. Reliability and validity were also evaluated.

**Results:**

The results indicate that the EQ-5D-5L surpasses the EQ-5D-3L in construct validity, informativity, detection precision, and discriminatory power. Consistency reliability is also better in the EQ-5D-5L than in the EQ-5D-3L. Both instrument versions maintained reliable levels of test–retest reliability.

**Conclusion:**

In patients with KOA undergoing UKA, the EQ-5D-5L has proven superior in measurement capabilities when compared with the EQ-5D-3L one-year post-operation. Thus, it is advised to utilize the EQ-5D-5L for ongoing assessments of quality of life in this specific group of patients.

## 1 Introduction

The EuroQol five dimensions questionnaire (EQ-5D), created by the EuroQol Group, incorporates five dimensions and is globally recognized as one of the foremost patient-reported outcome measures (PROMs) (1), extensively used in various countries. It employs distinct scoring algorithms across more than 20 nations (2, 3). This tool is essential for research in clinical and health services, economic analyses that compute cost per quality-adjusted life year, and has lately expanded its applications (4). Additionally, it provides an overarching evaluation of health-related quality of life and complements specific instruments for knee evaluations (5). The EQ-5D was originally available only in the EQ-5D-3L, which includes five dimensions (mobility, self-care, usual activities, pain/discomfort, and anxiety/depression), with each dimension having three functional levels (no problems, some problems, extreme problems). Building upon the EQ-5D-3L, the EQ-5D-5L was subsequently developed (6). The EQ-5D-5L includes five dimensions (mobility, self-care, usual activities, pain/discomfort, and anxiety/depression), with each dimension having five functional levels (no problems, slight problems, moderate problems, severe problems, extreme problems) (7, 8).

PROMs are increasingly popular in clinical assessments. To reduce patient burden while providing useful clinical information, clinicians should ensure the scales used are sensitive enough to changes and are also brief. The EQ-5D-3L has raised increasing doubts regarding its sensitivity to changes (5, 7, 9–12). Recent studies have indicated that the EQ-5D-5L, in comparison to the EQ-5D-3L, has shown improved sensitivity across various patient populations with different medical conditions. Zhu and colleagues (13) in the evaluation of EQ-5D across patients with breast cancer, colorectal cancer, or lung cancer, found that the EQ-5D-5L displayed improved performance in ceiling effects, reliability, and validity when compared to the EQ-5D-3L. In a study by Kontodimopoulos and colleagues (14), involving patients with rheumatoid arthritis, psoriatic arthritis, ankylosing spondylitis, and osteopenia/osteoporosis, the EQ-5D-5L demonstrated greater accuracy, consistency, and reliability compared to the EQ-5D-3L. For patients undergoing total knee replacement due to KOA, similar conclusions have been drawn regarding the responsiveness in preoperative and postoperative assessments (5, 10, 15–18), comparative studies on the construct validity of the EQ-5D-5L and EQ-5D-3L in this population have also shown that the EQ-5D-5L outperforms the EQ-5D-3L, for example, Greene and colleagues (10) along with Conner-Spady and colleagues (5) have established through their research that for individuals who have received total hip or knee replacements, the EQ-5D-5L offers improved construct validity compared to the EQ-5D-3L.

For patients undergoing UKA for unicompartmental osteoarthritis, the procedure is less invasive, with reduced bone loss and minimal blood loss. Compared to patients undergoing total knee arthroplasty, undergoing UKA patients experience a faster initial recovery and achieve better outcomes in functional results, pain assessment, revision rates, and complication incidence (19–21). Therefore, higher precision in assessment PROMs is required for evaluating this patient group. The measurement properties of both the EQ-5D-3L and EQ-5D-5L in patients undergoing UKA have already been demonstrated. However, only studies on the validity and responsiveness of individual versions exist. For example, Baryeh and colleagues (22) compared the relationship between patient satisfaction of UKA patients and preoperative and postoperative changes in EQ-5D-5L. Farrow and colleagues (23) compared the relationship between preoperative EQ-5D-3L scores and the extent of postoperative improvements in quality of life and joint function among patients who received total hip or knee replacements and those who underwent UKA.

Currently, no studies have compared the measurement properties of the two EQ-5D versions in patients undergoing UKA, and there is insufficient evidence to recommend either the EQ-5D-3L or EQ-5D-5L for assessing health status in this population. Therefore, this study aims to assess the measurement properties of both EQ-5D versions in evaluating one-year quality of life outcomes in patients post-UKA surgery.

## 2 Materials and methods

### 2.1 Data collection

In the medical records of the knee joint surgery department at the Honghui Hospital, Xi’an Jiaotong University, 402 patients aged 50 years or older who had undergone UKA and were one-year post-operation were selected. They were invited to complete questionnaires that incorporated both versions of EQ-5D and EQ-VAS, alongside general health-related measures like the Short Form-6 Dimension (SF-6D), and knee-specific PROMs, such as the Western Ontario and McMaster Universities Arthritis Index (WOMAC). Prior to questionnaire administration, the research objectives were clarified to the patients, and informed consent was secured. The sequence in which the two EQ-5D versions were presented was randomized, as was the order of domains within each EQ-5D in a subsequent retest. Exclusion criteria included illiteracy, lack of cognitive recognition, presence of severe chronic conditions such as malignant tumors, and cardiovascular or cerebrovascular diseases, along with psychiatric disorders. The study focused on data from patients who completed both EQ-5D versions for statistical purposes. Of the subjects approached, 231 individuals (57.5%) completed the questionnaires; 228 completed both versions. 96 respondents were randomly informed to complete a retest questionnaire 2 weeks later, and 56 (58%) completed the EQ-5D retest questionnaire.

The study adheres to the principles of the Declaration of Helsinki, and ethics approval was obtained from the medical ethics committee of Honghui Hospital, Xi’an Jiaotong University (No.202201039).

### 2.2 Instruments

The EQ-5D instrument encompasses five dimensions: mobility, self-care, usual activities, pain/discomfort, and anxiety/depression. The EQ-5D-3L articulates each dimension across three levels, depicting 243 distinct health states, usually denoted as health carriers from 11,111 (indicating perfect health) to 33,333 (representing the poorest health condition) (7, 9, 24). Conversely, the EQ-5D-5L details each dimension through five levels (7, 9), thereby delineating 3,125 distinct health states, conventionally reported from 11,111 (perfect health) to 55,555 (poorest health) (25). In addition to the EQ-5D-3L and EQ-5D-5L descriptive systems, the EQ-5D tool also includes a visual analog scale (VAS), EQ-VAS (2, 8), which is a separate component where health is self-rated on a 20-cm vertical scale, ranging from “Best imaginable health state” (100) to “Worst imaginable health state” (0) (8). Both the EQ-5D-3L and EQ-5D-5L are designed to be completed within 2 to 5 min (10). The Chinese value sets for both EQ-5D-3L and EQ-5D-5L have been developed, with the value range for EQ-5D-3L being [−0.149, 1.000] and for EQ-5D-5L being [−0.391, 1.000] (where 1 represents a state of perfect health) (26, 27).

The SF-6D is a universal PROMs, defined by six hierarchical health dimensions: physical functioning, role limitations, social functioning, pain, mental health, and vitality. Each dimension is divided into 4 to 6 levels (9). A six-digit code represents an SF-6D health state, derived by selecting a level from each dimension, commencing with physical functioning and culminating in vitality (9). The code 111,111 denotes optimal health, while 645,655 represents the least favorable health condition (28).

The WOMAC is a 24-item knee joint disease-specific functional measure that encompasses three domains: pain, stiffness, and physical function (29–31). Each of the 24 questions is rated using either a Likert 5-point scale or a 100-mm VAS, with scores ranging from “none or 0” to “extreme or 100.” In this investigation, the VAS from the Fajardo version was employed. Patients are instructed on VAS usage before answering the questions (30, 31). Section A scores range from 0 to 500, Section B from 0 to 200, and Section C from 0 to 1700 (30, 31). Higher scores reflect more pain, stiffness, and poorer physical function (30, 31).

### 2.3 Statistical analysis

To assess missing data, the collected dataset is analyzed to compare the two versions of EQ-5D, focusing only on samples with complete data for both versions in the statistical analysis. The evaluation includes examining the five items of EQ-5D, the index values, and the EQ-VAS. Shannon’s indices ( $H^{\prime }$ ) and evenness index ( $J^{\prime }$ ) are used to evaluate the informativity and discriminatory power of the two EQ-5D versions (32). Shannon index ( $H^{\prime }$ ) expresses the absolute amount of informativity captured in a system. In this formula, $H^{\prime }$ is calculated as follows:


$$H^{\prime }=-\overset{L}{\underset{i=1}{\sum }}p_{i}\log_{2}p_{i}$$

Where L denotes the number of response levels in a given dimension, and $p_{i}$ represents the proportion of observations at the i -th level ( i = 1, …, L). As $H^{\prime }$ increases, more information is captured.

$J^{\prime }$ is the evenness index, representing the ratio of the observed $H^{\prime }$ to the maximum possible $H_{\text{max}}$ . The formula for $J^{\prime }$ is:


$$J^{\prime }=\frac{H_{\text{max}}}{H^{\prime }}$$

The maximum amount of information is obtained when the responses are uniformly distributed across all levels. Under these conditions, $H^{\prime }$ reaches its maximum value $H_{\text{max}}$ (1.58 for EQ-5D-3L and 2.32 for EQ-5D-5L) (16).

$J^{\prime }$ is used to assess the uniformity of information distribution, ranging from 0 to 1, with values closer to 1 indicating a more even distribution of information (16).

Previous studies have demonstrated variations within specific dimensions of EQ-5D, where responses from the EQ-5D-3L deviate by two or more levels compared to the EQ-5D-5L. The ‘New’ response classification signifies a one-level difference between the questionnaire versions, whereas an ‘Inconsistent’ response is characterized by alterations spanning two or more severity levels between the assessments (28, 33). Thus, the magnitude of inconsistency spans from 1 (representing a discrepancy of two levels) to 3 (indicating a discrepancy of four levels) (11). Calculations are performed to determine both the proportion of consistent versus inconsistent response pairs between the EQ-5D-3L and EQ-5D-5L and the average magnitude of these inconsistencies (11).

Compare the SDC for groups and individuals between the two versions of EQ-5D. The SDC for groups represents the minimum change necessary to identify a true difference within a population, whereas the SDC for individuals reflects the smallest detectable change for an individual. By comparing the two versions of EQ-5D, this approach enables assessment of the sensitivity of each version in detecting clinically meaningful changes. The SDC_groups_ and SDC_individuals_, representing the smallest change in value for a scale that can be considered an actual change rather than a measurement error, are calculated using the following formula: SDC_individuals_ = 1.96· √ 2·SEM, and SDC_group_ = ${SDC}_{\mathrm{individuals}}/\surd n$ . Where SEM = SD· √ (1-R), SD = the sample standard deviation, R = the calculated Intraclass correlation coefficient (ICC), and n is the sample size (34).

To assess the test–retest reliability of the EQ-5D tools, ICC are commonly employed, as they gauge the level of agreement or score stability across time. Test–retest reliability examines the consistency with which the EQ-5D-3L and EQ-5D-5L instruments produce similar outcomes when administered to the same patient group at two distinct time points (35). An ICC value of 0.70 or above is considered indicative of excellent test–retest reliability (35).

The internal consistency of the EQ-5D-3L and EQ-5D-5L versions is independently evaluated for each version through the application of Cronbach’s alpha, which serves as a standard metric for assessing internal reliability. This coefficient measures the extent to which the items within each version align as a cohesive group, thereby representing the proportion of variance in scores that can be attributed to differences among individuals rather than inconsistencies within the instrument. A comparative analysis of the Cronbach’s alpha values between the two versions enables an assessment of their respective internal consistency (36). A Cronbach’s alpha value above 0.7 denotes acceptable consistency, whereas values below 0.7 suggest poor consistency (1).

According to the Consensus-based Standards for the Selection of Health Measurement Instruments (35), examine the construct validity of both iterations of EQ-5D against the EQ-VAS, SF-6D, and WOMAC by means of hypothesis testing, juxtaposing the scores [assessing hypotheses via Spearman’s correlation (Rho)] (16). The magnitudes of these coefficients are interpreted as small correlation (Rho < 0.20), moderate correlation (0.20 ≤ Rho < 0.50), or high correlation (Rho ≥ 0.50). A comparison of the ceiling effects of the two versions is conducted. Given the focus on the postoperative recovery period in this survey, it is expected that there will be more ceiling effects and fewer floor effects. However, comparing the proportion of reduction in ceiling effects remains meaningful.

All statistical analyses were conducted using IBM SPSS Statistics version R26.0.0.0.

## 3 Results

### 3.1 Data quality

Of the 402 patients who received invitations, 231 respondents (57.5%) completed the survey. Table 1 presents the characteristics of the 228 respondents who completed both versions of EQ-5D. According to widely accepted and recommended measurement standards (37), the sample size is adequate, meeting the Consensus-based Standards for the Selection of Health Measurement Instruments guidelines, which recommend at least 100 participants for studies assessing construct validity. Of the 96 patients who were randomly informed, 56 (58.3%) completed the retest questionnaire for both versions of EQ-5D. Table 1 presents the patient characteristics.

**Table 1:** Patient characteristics.

Variable	%
Age ≥ 60	178 (78.1%)
Female	164 (71.9%)
Male	64 (28.1%)
Years disease duration	
Years ≤ 5	66 (28.9%)
5 < Years ≤ 10	102 (44.8%)
Years > 10	60 (26.3%)
Surgery joint side	
Right	107 (46.9%)
Left	121 (53.1%)
Educational level	
Primary or secondary	210 (92.1%)
Tertiary	18 (7.9%)
Living status	
Alone	6 (2.6%)
With spouse	144 (63.2%)
With children	73 (32.2%)
BMI (kg/m^2^)	
BMI ≤ 23.9 (underweight or healthy)	64 (28.1%)
23.9 < BMI ≤ 27.9 (overweight)	107 (45.0%)
BMI > 27.9 (obese)	57 (25.0%)
Occupation	
Heavy manual worker	17 (7.4%)
Light manual worker	17 (7.4%)
Agricultural	24 (10.5%)
Retired	170 (74.6%)

### 3.2 Informativity and discriminatory power

**Figure 1:**
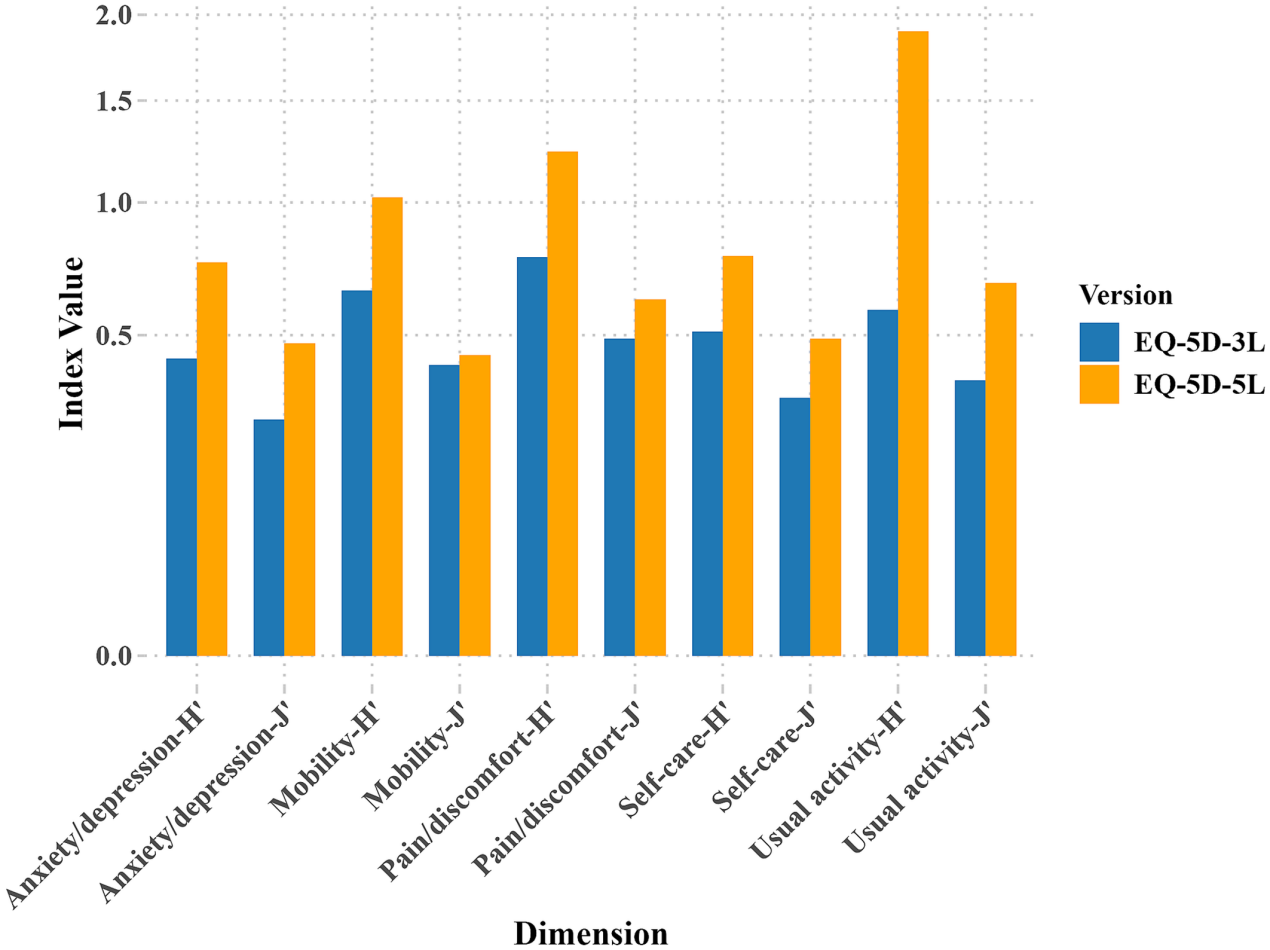
Informativity and discriminatory power of EQ-5D: results for $H^{\prime }$ and $J^{\prime }$ .

Figure 1 shows that Shannon’s $H^{\prime }$ ranges from 0.429 (Anxiety/depression) to 0.773 (Pain/discomfort) for the EQ-5D-3L and from 0.752 (Anxiety/depression) to 1.898 (Usual activity) for the EQ-5D-5L. The $J^{\prime }$ ranges from 0.271 (Anxiety/depression) to 0.489 (Pain/discomfort) for EQ-5D-3L and from 0.475 (Anxiety/depression) to 0.676 (Usual activity) for EQ-5D-5L. As shown in Figure 1, overall, in terms of informativity and discriminatory power across all dimensions, the EQ-5D-5L consistently demonstrates higher H′ and J′ values compared to the EQ-5D-3L, indicating that the EQ-5D-5L provides greater informativity and discriminatory power than the EQ-5D-3L.

### 3.3 Re-allocation of attributes and inconsistency

**Figure 2:**
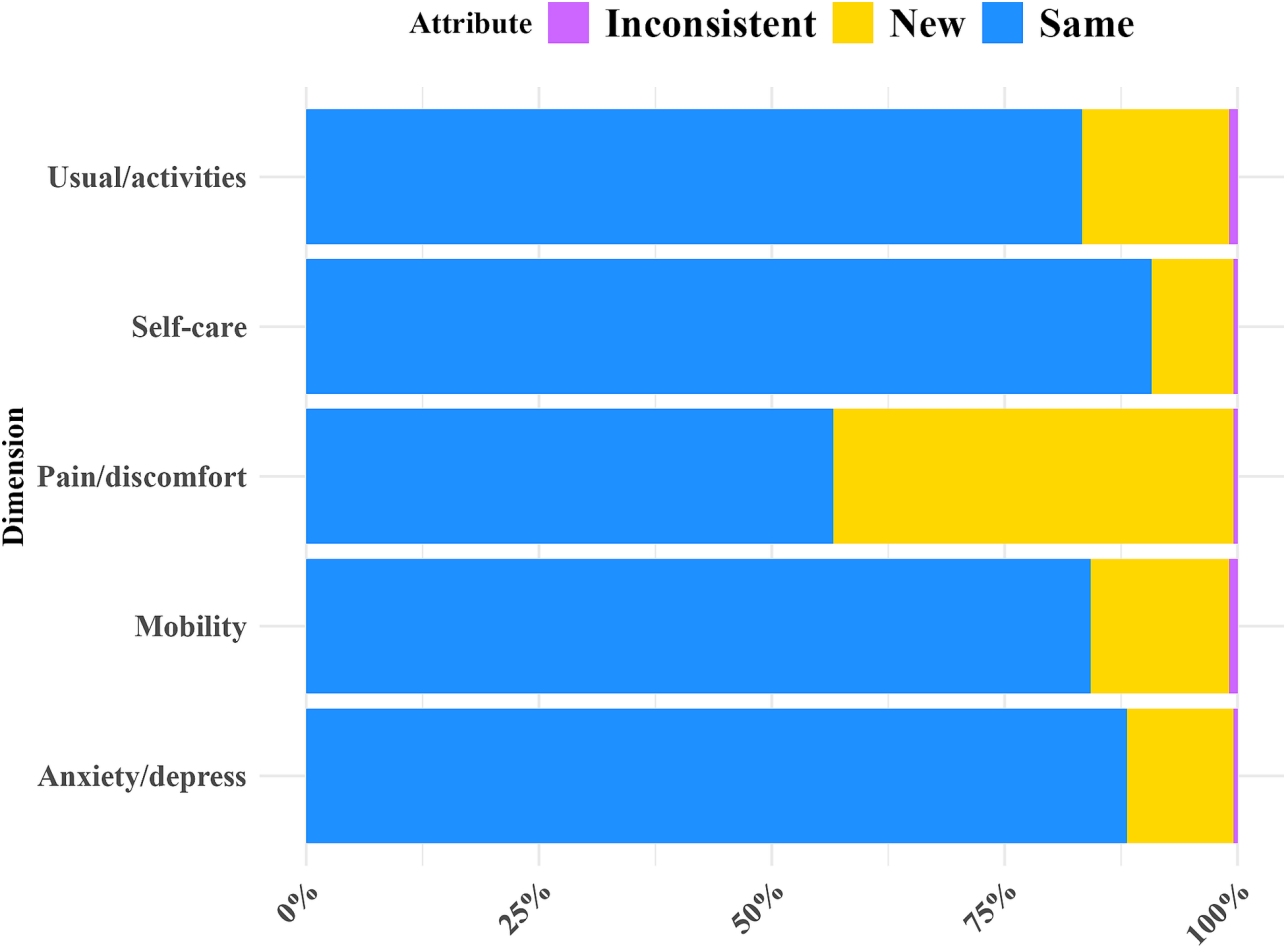
Redistribution properties from EQ-5D-3L to EQ-5D-5L responses.

Table 2 shows the redistribution properties of responses from EQ-5D-3L to EQ-5D-5L, along with the number of consistent and inconsistent response pairs. The table provides a cross-tabulation of dimension scores. In the reallocation characteristics of responses from the EQ-5D-3L to the EQ-5D-5L, there were 228 evaluations of both EQ-5D-3L and EQ-5D-5L, producing 1,128 response pairs, among which there were 12 inconsistent responses appearing in 12 out of the 228 evaluations (5.3%). Two respondents selected “1” indicating no problems in the EQ-5D-3L Usual activities dimension, but selected “3” indicating moderate problems in the EQ-5D-5L Usual activities. Six respondents chose “2” indicating some problems in the EQ-5D-3L Mobility dimension, but selected “1” indicating no problems in the EQ-5D-5L Mobility. Two respondents selected “2” in the EQ-5D-3L Anxiety/depression dimension, but selected “1” indicating no problems in the EQ-5D-5L Anxiety/depression. Two respondents chose “3” indicating extreme problems in the EQ-5D-3L Pain/discomfort dimension, but selected “2” indicating moderate problems in the EQ-5D-5L Pain/discomfort. The rate of inconsistency in responses from EQ-5D-3L to EQ-5D-5L: Mobility and Self-care had an inconsistency rate of 2.6%. Usual activities, Pain/discomfort, and Anxiety/depression had an inconsistency rate of 0.9% (Figure 2).

**Table 2:** Redistribution properties from EQ-5D-3L to EQ-5D-5L responses.

		EQ-5D-5L					Consistent		Inconsistent	
EQ-5D-3L Dimensions		1	2	3	4	5	n	%	n	%
1	Mobility	168	22	**0**	**0**	**0**	190	100%	0	0%
	Self-care	192	10	**0**	**0**	**0**	202	100%	0	0%
	Usual activities	170	28	**2**	**0**	**0**	198	99.0%	2	1.0%
	Pain/discomfort	96	90	**0**	**0**	**0**	186	100%	0	0%
	Anxiety/depression	186	22	**0**	**0**	**0**	208	100%	0	0%
2	Mobility	**6**	18	12	2	**0**	32	84.2%	6	15.8%
	Self-care	**0**	20	6	0	**0**	26	100%	0	0%
	Usual activities	**0**	20	6	0	**0**	26	100%	0	0%
	Pain/discomfort	**0**	32	6	0	**0**	38	100%	0	0%
	Anxiety/depression	**2**	14	4	0	**0**	18	90.0%	2	10.0%
3	Mobility	**0**	**0**	**0**	0	0	0	0%	0	0%
	Self-care	**0**	**0**	**0**	0	0	0	0%	0	0%
	Usual activities	**0**	**0**	**0**	2	0	2	100%	0	0%
	Pain/discomfort	**0**	**0**	**2**	2	0	2	50.0%	2	50.0%
	Anxiety/depression	**0**	**0**	**0**	0	0	0	0%	0	0%
Consistent response pair	n	812	276	34	6	0	1,128	98.9%	–	–
	%	71.2%	24.2%	3.0%	0.5%	0%				
Inconsistent response pair	n	8	0	4	0	0	–	–	12	1.1%
	%	0.7%	0%	0.4%	0%	0%				
Total response pair	n	820	276	38	6	0	1,140 (100%)			

Inconsistencies (marked in bold) are defined as differences of more than one between EQ-5D-3L and EQ-5D-5L response categories.

Redistribution properties from EQ-5D-3L to EQ-5D-5L responses of *n* = 228 EQ-5D-3L and EQ-5D-5L assessments, generating 1,128 response pairs 12 inconsistent pairs arise out of 12 of 228 assessments (5.3%).

### 3.4 Reliability

**Figure 3:**
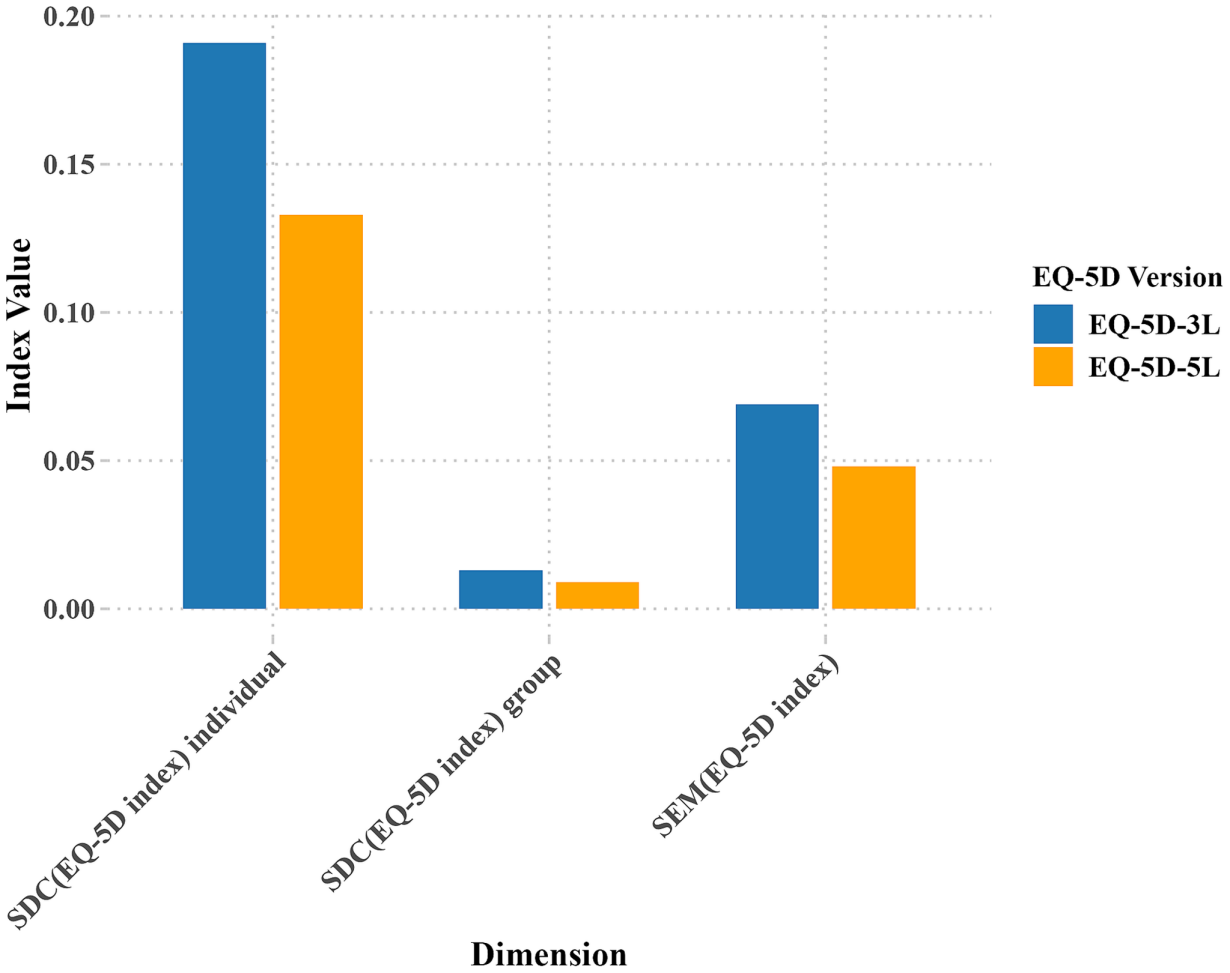
Comparison of EQ-5D index SDC and SEM results.

**Figure 4:**
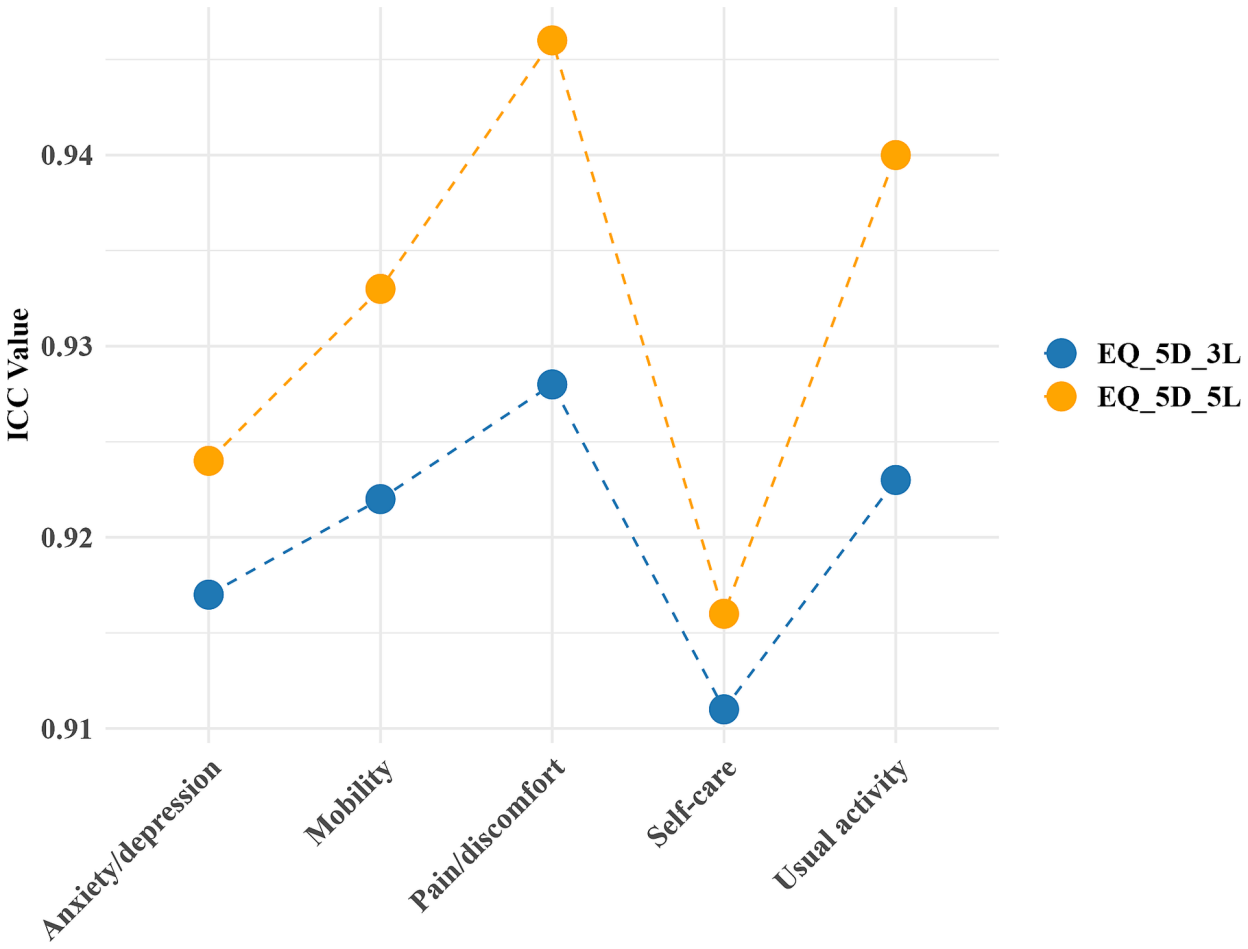
The ICC for test–retest reliability of EQ-5D.

Figure 3 shows that the SDC at the individual level is 0.191 for EQ-5D-3L and 0.133 for EQ-5D-5L, while at the group level, it is 0.013 for EQ-5D-3L and 0.009 for EQ-5D-5L. The measurement accuracy of EQ-5D-5L is higher than that of EQ-5D-3L. Figure 4 shows the test–retest reliability of EQ-5D, evaluated according to recommended and widely used measurement standards (ICC > 0.7) (37), indicating good test–retest reliability. The Cronbach’s alpha for within-group correlation is 0.881 for EQ-5D-5L and 0.710 for EQ-5D-3L.

### 3.5 Validity

**Figure 5:**
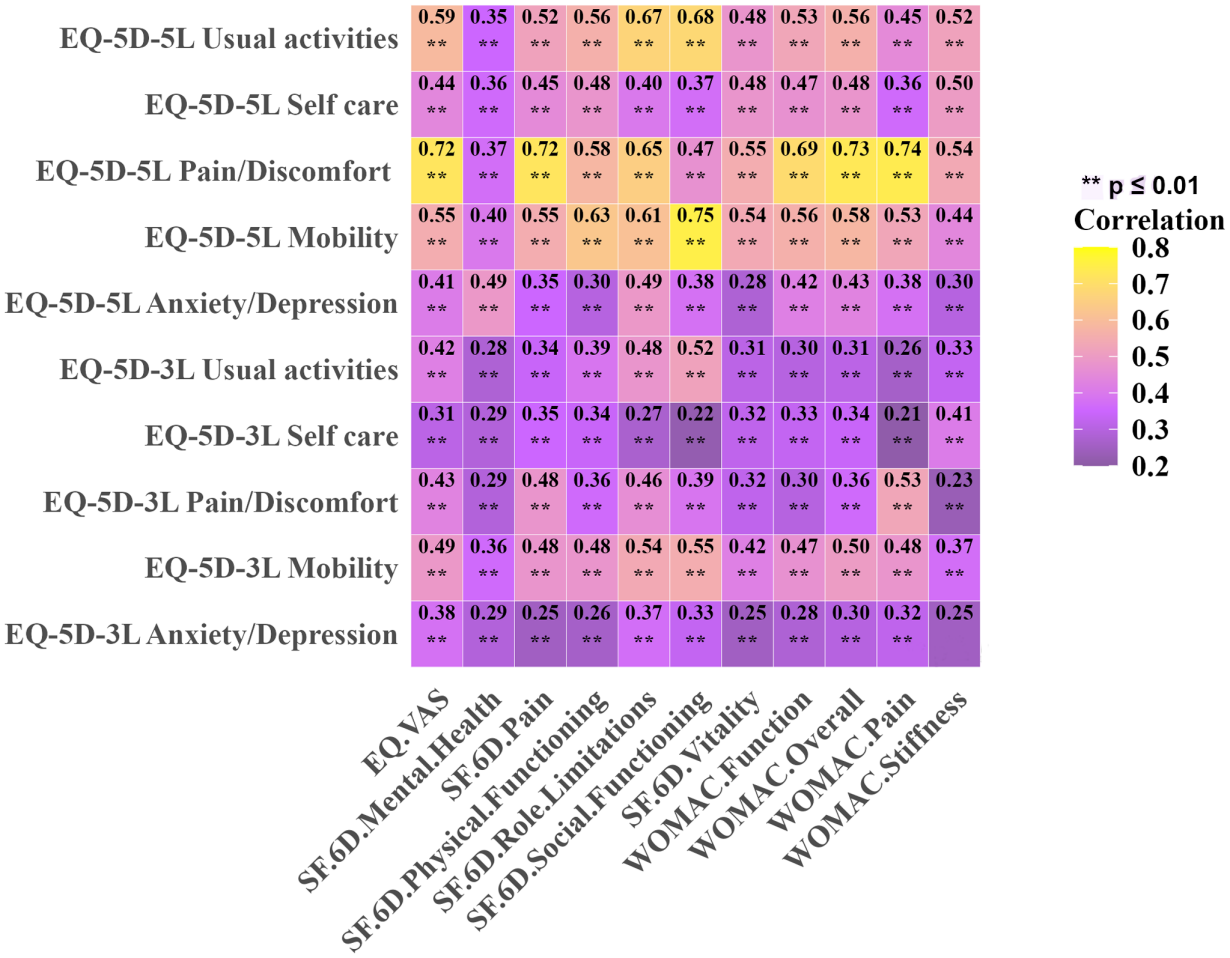
Spearman’s correlation for EQ-5D at the dimension level with WOMAC, SF-6D, and EQ-VAS. **p ≤ 0.01.

Figure 5 shows the correlations between EQ-5D-3L and EQ-5D-5L dimensions, index scores, WOMAC, SF-6D, and EQ-VAS scores. All correlations were statistically significant with *p* ≤ 0.01. The Spearman correlation coefficients between the EQ-5D-3L dimensions and EQ-VAS range from 0.26 to 0.49, and for the EQ-5D-5L dimensions, from 0.41 to 0.72. Correspondingly, the correlation between the EQ-5D index and EQ-VAS are 0.63 and 0.77, respectively. Compared to the EQ-5D-3L, all dimensions and index scores of the EQ-5D-5L show a high correlation with EQ-VAS. In terms of Spearman correlation coefficients with SF-6D, the EQ-5D-5L index and dimensions generally have higher correlations than the EQ-5D-3L, ranging from 0.30 to 0.72 for EQ-5D-5L, compared to 0.22 to 0.55 for EQ-5D-3L. In the correlation analysis with WOMAC, the EQ-5D-5L dimensions generally showed higher correlations than the EQ-5D-3L dimensions. The EQ-5D-5L index score correlated more strongly with WOMAC than the EQ-5D-3L index, which showed moderate correlations with stiffness and function (0.48 and 0.49 respectively), and was highly correlated with pain and the overall score (0.50 and 0.53 respectively). The EQ-5D-5L index was highly correlated with all WOMAC dimensions (from 0.62 to 0.76). Overall, the EQ-5D-5L demonstrates higher correlations with both the overall and specific domain scores of WOMAC and SF-6D compared to the EQ-5D-3L. The same is true for their correlation with EQ-VAS. This indicates that the EQ-5D-5L has better construct validity. Compared to the EQ-5D-3L index, the EQ-5D-5L index demonstrates a 24.6% reduction in the ceiling effect.

## 4 Discussion

This study compares the psychometric properties of the EQ-5D-5L and EQ-5D-3L in KOA undergoing UKA. Compared to the EQ-5D-3L, the EQ-5D-5L showed superior performance across all dimensions in terms of absolute informativity ( $H^{\prime }$ ) and the uniformity of information distribution ( $J^{\prime }$ ). Consequently, the EQ-5D-5L exhibited greater informativity and discriminatory power. Both the EQ-5D-3L and EQ-5D-5L demonstrated good reliability. In terms of validity, the EQ-5D-5L also outperformed the EQ-5D-3L. Our study suggests that the EQ-5D-5L is recommended for assessing quality of life in assessing one-year quality of life outcomes for patients with KOA undergoing UKA.

Current research indicates that the Shannon’s $H^{\prime }$ index for the dimensions of EQ-5D-3L and EQ-5D-5L ranges from 0.429 (Anxiety/Depression) to 0.773 (Pain/Discomfort) and from 0.752 (Anxiety/Depression) to 1.898 (Usual Activities), respectively. The $J^{\prime }$ index for the EQ-5D-3L and EQ-5D-5L dimensions ranges from 0.271 (Anxiety/Depression) to 0.489 (Pain/Discomfort) and from 0.475 (Anxiety/Depression) to 0.676 (Usual Activities). Overall, the EQ-5D-5L version exhibits greater Informativity and discriminatory power than the EQ-5D-3L. Compared to the EQ-5D-3L, the EQ-5D-5L adds two levels per dimension, expanding its sensitivity and applicability. This enhancement results in greater informativity and improved discriminatory power. This finding showing a strong consistency with the findings reported in other studies. Conner-Spady and colleagues (5) examined the informativity and discriminatory power of the EQ-5D in a cohort of 176 patients undergoing hip or knee replacement surgery due to osteoarthritis. Their findings showed that the EQ-5D-5L demonstrated greater informativity and improved discriminatory power compared to the EQ-5D-3L. Similarly, Michalowsky and colleagues (11) compared the psychometric properties of the EQ-5D-3L and EQ-5D-5L based on proxy ratings by informal caregivers and health professionals for patients with dementia. Their results also indicated that the EQ-5D-5L exhibited greater informativity and superior discriminatory power relative to the EQ-5D-3L.

According to the recommended and widely applied measurement standards (37), both versions of the EQ-5D have good test–retest reliability. No indications of consistent disparities between the initial and subsequent assessment outcomes are observed. The Cronbach’s alpha for within-group correlation is 0.881 for EQ-5D-5L and 0.710 for EQ-5D-3L, indicating good internal consistency for both versions of EQ-5D (Cronbach’s alpha >0.7). The SDC at the group level for the EQ-5D-3L and EQ-5D-5L are 0.133 and 0.009, respectively, and at the individual level, they are 0.191 and 0.013, respectively. The EQ-5D-5L’s detection accuracy is higher than that of the EQ-5D-3L. These results are consistent with previous research by Garratt and colleagues (16) who examined patients undergoing surgical fixation for closed ankle fractures. In their study, the SDC values for the EQ-5D-5L index were reported as 0.02 at the group level and 0.20 at the individual level, underscoring the EQ-5D-5L’s greater sensitivity for detecting subtle changes in health status across both levels. Both studies demonstrate the sensitivity advantage of the EQ-5D-5L across different health states and patient populations. This finding further validates the reliability and applicability of the EQ-5D-5L in detecting subtle health changes across diverse populations.

Our findings indicated that the EQ-5D-5L demonstrated stronger construct validity coefficients with other health-related measures compared to the EQ-5D-3L, highlighting its enhanced sensitivity in evaluating postoperative quality of life. Similarly, Conner-Spady and colleagues (5) assessed the construct validity of the EQ-5D among 176 patients undergoing hip or knee replacement for osteoarthritis and found that the EQ-5D-5L showed better alignment with related health measures than the EQ-5D-3L, consistent with our findings. Greene and colleagues (10) further examined a cohort of total hip arthroplasty patients, observing that the EQ-5D-5L provided a more refined assessment of health-related quality of life compared to the EQ-5D-3L. Eneqvist and colleagues (38) in their study of Swedish total hip replacement patients, also confirmed that the EQ-5D-5L demonstrated superior construct validity coefficients.

Given the survey focus on one-year follow-up, it is expected that a significant portion of participants will reach optimal levels of health status. In this study, the ceiling effect observed for the EQ-5D-5L index decreased by 24.6% compared to the EQ-5D-3L index. Although systematic reviews comparing the EQ-5D-3L and EQ-5D-5L do not include populations with one-year follow-up, they have identified more substantial differences in ceiling effects, with the highest disparities reaching up to 30% (32). Recent findings have indicated a noteworthy decrease in the ceiling effect of the EQ-5D-5L when compared to the EQ-5D-3L in multiple recent investigations (5, 10, 11, 18, 39).

This study’s focus on the postoperative recovery phase likely contributed to an elevated ceiling effect compared to other stages of disease progression. A key limitation is the absence of responsiveness assessments; future studies should include these to assess the instruments’ sensitivity to change over time. As a single-center study with exclusively Chinese participants, however, the generalizability of these findings may be limited. Conducting research across multiple centers and diverse ethnic populations could provide a more comprehensive view of EQ-5D-3L and EQ-5D-5L performance in varied healthcare contexts. Additionally, extending follow-up beyond one year could further elucidate the EQ-5D’s capacity to reflect one-year quality of life changes. Finally, including other PROMs alongside EQ-5D would allow for a multidimensional assessment of health-related quality of life, and could help validate EQ-5D’s construct validity through comparisons with other established health-related quality of life instruments.

## 5 Conclusion

Overall, in patients with KOA undergoing UKA, one-year post-operation, both versions were within acceptable performance ranges, with the EQ-5D-5L demonstrating superior performance. Therefore, the EQ-5D-5L is recommended for assessing quality of life in this patient group.

## Data Availability

The raw data supporting the conclusions of this article will be made available by the authors, without undue reservation.
