# Supplementary material for: Comparison of the EQ-5D-3L and EQ-5D-5L instruments in patients undergoing unicompartmental knee arthroplasty
Source: Front Med (Lausanne). 2025 Jan 6;11:1451979. doi: 10.3389/fmed.2024.1451979 (PMC11744716; doi:10.3389/fmed.2024.1451979)
Supplement: Supplementary file 1 [file Table_1.DOCX]

EQ-5D-5L

| 在每个标题下,请在能最恰当地描述您今天的健康状况的一个方格上打“√” |  |
| --- | --- |
| **行动能力** |  |
| 我四处走动没有困难 |  |
| 我四处走动有一点困难 |  |
| 我四处走动有中度的困难 |  |
| 我四处走动有严重的困难 |  |
| 我无法四处走动 |  |
|  |  |
| **自我照顾** |  |
| 我自己洗澡或穿衣没有困难 |  |
| 我自己洗澡或穿衣有一点困难 |  |
| 我自己洗澡或穿衣有中度的困难 |  |
| 我自己洗澡或穿衣有严重的困难 |  |
| 我无法自己洗澡或穿衣 |  |
|  |  |
| **日常活动(如工作、学习、家务、家庭或休闲活动)** |  |
| 我进行日常活动没有困难 |  |
| 我进行日常活动有一点困难 |  |
| 我进行日常活动有中度的困难 |  |
| 我进行日常活动有严重的困难 |  |
| 我无法进行日常活动 |  |
|  |  |
| **疼痛或不舒服** |  |
| 我没有疼痛或不舒服 |  |
| 我有一点疼痛或不舒服 |  |
| 我有中度的疼痛或不舒服 |  |
| 我有严重的疼痛或不舒服 |  |
| 我有非常严重的疼痛或不舒服 |  |
|  |  |
| **焦虑或沮丧** |  |
| 我没有焦虑或沮丧 |  |
| 我有一点焦虑或沮丧 |  |
| 我有中度的焦虑或沮丧 |  |
| 我有严重的焦虑或沮丧 |  |
| 我有非常严重的焦虑或沮丧 |  |

EQ-5D-3L

| 在每个标题下, 请在能最恰当地描述您今天的健康状况的一个方格上打 “√”。 |  |
| --- | --- |
| **行动能力** |  |
| 我可以四处走动, 没有任何困难 |  |
| 我四处走动有些不方便 |  |
| 我不能下床活动 |  |
|  |  |
| **自己照顾自己** |  |
| 我能自己照顾自己, 没有任何困难 |  |
| 我在洗脸、刷牙、洗澡或穿衣方面有些困难 |  |
| 我无法自己洗脸、刷牙、洗澡或穿衣 |  |
|  |  |
| **日常活动(如工作、学习、家务、家庭或休闲活动)** |  |
| 我能进行日常活动,没有任何困难 |  |
| 我在进行日常活动方面有些困难 |  |
| 我无法进行日常活动 |  |
|  |  |
| **疼痛 / 不舒服** |  |
| 我没有任何疼痛/不舒服 |  |
| 我觉得中度疼痛/不舒服 |  |
| 我觉得极度疼痛/不舒服 |  |
|  |  |
| **焦虑(如紧张、担心、不安等等)/抑郁(如做事情缺乏兴趣、没乐趣、提不起精神等等)** |  |
| 我不觉得焦虑或抑郁 |  |
| 我觉得中度焦虑或抑郁 |  |
| 我觉得极度焦虑或抑郁 |  |
